# Altered functional organization within the insular cortex in adult males with high-functioning autism spectrum disorder: evidence from connectivity-based parcellation

**DOI:** 10.1186/s13229-016-0106-8

**Published:** 2016-10-05

**Authors:** Takashi Yamada, Takashi Itahashi, Motoaki Nakamura, Hiromi Watanabe, Miho Kuroda, Haruhisa Ohta, Chieko Kanai, Nobumasa Kato, Ryu-ichiro Hashimoto

**Affiliations:** 1 Medical Institute of Developmental Disabilities Research, Showa University, 6-11-11 Kita-karasuyama, Setagaya-ku, Tokyo, Japan; 2 ATR Brain Information Communication Research Laboratory Group, 2-2-2 Hikaridai, Seika-cho, Sorakugun, Kyoto, Japan; 3 Kinko Hospital, Kanagawa Psychiatric Center, 2-5-1 Serigaya, Yokohama, Kanagawa Japan; 4 Child Mental Health-care Center, Fukushima University, 1 Kanayagawa, Fukusima-shi, Fukushima, Japan; 5 Department of Child Neuropsychiatry, Graduate School of Medicine, The University of Tokyo, 7-3-1 Hongou, Bunkyo-ku, Tokyo, Japan; 6 Department of Language Sciences, Graduate School of Humanities, Tokyo Metropolitan University, 1-1 Minami-Osawa, Hachioji-shi, Tokyo, Japan; 7 Research Center for Language, Brain and Genetics, Tokyo Metropolitan University, 1-1 Minami-Osawa, Hachioji-shi, Tokyo, Japan

**Keywords:** Autism spectrum disorder, Resting-state functional magnetic resonance imaging, Insula, Connectivity-based functional parcellation

## Abstract

**Background:**

The insular cortex comprises multiple functionally differentiated sub-regions, each of which has different patterns of connectivity with other brain regions. Such diverse connectivity patterns are thought to underlie a wide range of insular functions, including cognitive, affective, and sensorimotor processing, many of which are abnormal in autism spectrum disorder (ASD). Although past neuroimaging studies of ASD have shown structural and functional abnormalities in the insula, possible alterations in the sub-regional organization of the insula and the functional characteristics of each sub-region have not been examined in the ASD brain.

**Methods:**

Resting-state functional magnetic resonance imaging (rs-fMRI) data were acquired from 36 adult males with ASD and 38 matched typically developed (TD) controls. A data-driven clustering analysis was applied to rs-fMRI data of voxels in the left and right insula to automatically group voxels with similar intrinsic connectivity pattern into a cluster. After determining the optimal number of clusters based on information theoretic measures of variation of information and mutual information, functional parcellation patterns in both the left and the right insula were compared between the TD and ASD groups. Furthermore, functional profiles of each sub-region were meta-analytically decoded using Neurosynth and were compared between the groups.

**Results:**

We observed notable alterations in the anterior sector of the left insula and the middle ventral sub-region of the right insula in the ASD brain. Meta-analytic decoding revealed that whereas the anterior sector of the left insula contained two functionally differentiated sub-regions for cognitive, sensorimotor, and emotional/affective functions in TD brain, only a single functional cluster for cognitive and sensorimotor functions was identified in the anterior sector in the ASD brain. In the right insula, the middle ventral sub-region, which is primarily specialized for sensory- and auditory-related functions, showed a significant volumetric increase in the ASD brain compared with the TD brain.

**Conclusions:**

The results indicate an altered organization of sub-regions in specific parts of the left and right insula of the ASD brain. The alterations in the left and right insula may constitute neural substrates underlying abnormalities in emotional/affective and sensory functions in ASD.

**Electronic supplementary material:**

The online version of this article (doi:10.1186/s13229-016-0106-8) contains supplementary material, which is available to authorized users.

## Background

Autism spectrum disorder (ASD) has been increasingly conceptualized as a disease of large-scale brain networks [1]. Among multiple nodes that constitute the brain’s networks, several brain regions have emerged as key structures that particularly contribute to abnormal functionalities in the ASD brain. The insular cortex is one such brain region, whose structural and functional abnormalities are frequently reported in the neuroimaging literature of ASD. Structurally, alterations of the gray matter (GM) volume have been identified in the anterior and posterior parts of the right insula in adult ASD [2–4]. Functionally, a comprehensive meta-analysis of functional imaging studies has revealed hypoactivation in the right anterior insula during various social tasks including face recognition and mentalizing [5]. Regarding connectivity, previous resting-state fMRI studies of adolescent and adult ASD have shown reduced functional connectivity (FC) of the anterior, middle, and posterior insula with distant brain regions, including the amygdala and the somatosensory cortex [6–8]. Convergent evidence from these structural, functional, and FC studies strongly prompts more detailed investigations of the insula to advance our understanding of the neural substrates of ASD.

Recent progress in anatomical studies has enabled more finely grained investigations into the neural organization of the insula. Traditionally, the insula has been divided into the following three regions: (1) anterior agranular, (2) posterior granular, and (3) precentral dysgranular [9–11]. However, a recent histological study has identified at least three distinct regions within the posterior insula alone [12], indicating the presence of a more fine-grained subdivision of this brain region. Similarly, FC analysis using fMRI data has led to recent progress in understanding the functional anatomy of the insular cortex. The rationale behind the FC approach is that the connectivity pattern of a brain region is a significant determinant of its functional role [13, 14]. FC patterns of the insular cortex have been mainly investigated using resting-state FC analyses or meta-analyses of co-activation patterns obtained in task-based fMRI studies. Using data-driven clustering methods in which voxels with similar FC patterns were grouped into a functional unit (parcel), Cauda and colleagues have identified two sub-regions of the ventral-anterior and dorsal-posterior insula based on resting-state FC [15] and co-activation patterns obtained in task-based fMRI studies [16]. On the other hand, a more recent study using data-driven clustering analysis of resting-state FC data supported the tripartite subdivision into dorsal-anterior, ventral-anterior, and posterior regions [17]. Based on the fact that each sub-region has a distinct FC pattern, this study used a meta-analytic co-activation tool (Neurosynth) and succeeded in decoding the functional profiles of insular sub-regions using reverse inference from the FC patterns seeded from each sub-region [18]. These studies demonstrate the utility of data-driven FC-based clustering methods in the study of insular functional organization, particularly when they are used in combination with meta-analytical tools such as the Neurosynth framework.

The aforementioned studies reporting a functional parcellation of the insula confirm the traditional cytoarchitecture-based subdivision scheme of this brain region. However, given more recent evidence indicating an even finer subdivision within the posterior insula, the data-driven FC clustering approach is expected to lead to a more finely grained functional parcellation of this brain region. Indeed, Kelly and colleagues gradually increased the number of parcels in the insula starting at two and examined changes in functional parcellation patterns using resting-state FC data, gray matter structural images, and a task-based fMRI co-activation map [19]. They observed consistent patterns of fine-scale functional parcellation across these three different modalities when using up to nine parcels. Comparing parcellation patterns at multiple scale levels (coarse vs. fine subdivisions) revealed that insular sub-regions are organized in a pseudo-hierarchical fashion, such that an increment in the number of subdivisions results in a finer division of an already existing cluster rather than generating a completely new parcellation pattern. These results indicate that the insular cortex is organized at multiple spatial scales and that finer parcellation patterns may offer additional insights into the functional organization of this structure.

In the present fMRI study, we examined the functional organization of the insula in adult males with ASD by applying the data-driven clustering method to resting-state FC data. Although localized alterations in insular gray matter volume, cortical activation, and FC have previously been reported in neuroimaging studies of ASD [6–8], to our knowledge, no study has examined the possibility of an altered organization of functionally heterogeneous insular sub-regions in the ASD brain. In this study, we first examined whether the functional organization of the insula is altered in subjects with ASD compared with typically developed (TD) controls. When we detected significant alterations, we then attempted to identify functions of the affected sub-regions by applying a meta-analytical decoding tool [18] to FC patterns of insular sub-regions. In this series of analyses, we needed to determine the optimal number of clusters for parcellation. Given previous findings indicating multiple spatial scales of insular organization [19], we expected that finer parcellation schemes would be more sensitive to subtle but significant functional alteration than coarser subdivisions adopted by traditional bi- or tripartite schemes.

## Methods

### Participants

Thirty-six high-functioning adult males with ASD and 38 age-matched TD males participated in this study. The diagnostic procedure to identify patients with ASD was the same as in our previous studies [20–22]. Briefly, an experienced psychiatrist and a clinical psychologist independently interviewed the patients (together with their caregivers when available) for approximately 3 h regarding their developmental history, present illness, life history, and family history. The diagnosis of ASD was made only when there was a consensus between the psychiatrist and clinical psychologist based on the criteria of the Diagnostic and Statistical Manual of Mental Disorders, Fourth Edition (DSM-IV-TR) (American Psychiatric, 2000). In addition, the diagnosis was reconfirmed after at least a 2-month of follow-up period. TD subjects were recruited through advertisements and acquaintances. None of the TD participants reported any severe medical problems or a neurological or psychiatric history. Except for six TD participants, participants completed the Japanese version of the autism spectrum quotient (AQ) test [23]. Table 1 shows detailed demographic information for the study participants.

**Table 1:** The demographic data for the participants

	TD (*n* = 38)			ASD (*n* = 36)			Statistics	
	Mean	SD	Range	Mean	SD	Range	df	*P* value
Age (years)	32.5	7.3	19–47	29.9	7.1	19–46	72	0.14
Full-scale IQ	109.5	8.4	87.5–119.8	106.2	13.9	83–134	72	0.23
Handedness	92.7	20.3	5.3–100	87.9	24.5	5.9–100	72	0.37
AQ score	15.5	5.6	7–30	36.3	4.9	23–43	66	<0.001
ADOS								
Total				12.8	3.8	5–21		
Communication				4.2	1.7	2–8		
Social reciprocity				8.6	2.8	3–12		

*Note*: WAIS-III or -R was administrated to all participants with ASD, and the IQ score was estimated for all TDs based on JART. The AQ score was collected from 32 TDs and all participants with ASD

We confirmed that all of the participants were right-handed using the Edinburgh Handedness Inventory [24]. The intelligence quotient (IQ) scores of all participants with ASD were evaluated using either the Wechsler Adult Intelligence Scale-Third Edition (WAIS-III) or the WAIS-Revised (WAIS-R), while those of TD subjects were estimated using a Japanese version of the National Adult Reading Test (JART) [25]. There were no significant differences between the two groups in age or IQ scores (both *p* > 0.1). Out of 36 participants with ASD, 24 individuals underwent either (1) Diagnostic Interview for Social and Communicative Disorders (DISCO) only (*n* = 5), or (2) Autism Diagnostic Observation Schedule (ADOS) only (*n* = 11), or (3) both (*n* = 8). The remaining 12 ASD participants did not undergo either of the auxiliary diagnostic tool of DISCO or ADOS. All of the five participants who underwent only the DISCO satisfied the DISCO diagnostic criteria for ASD. Out of the 11 participants who underwent only the ADOS, ten satisfied the ADOS diagnostic criteria for ASD, while one participant had a total ADOS score that was lower than the cut-off score (6 < 7); however, the same subject satisfied the cut-off scores for the “communication” and “social reciprocity” subscales of the ADOS (“communication”: 2 ≥ 2, “social reciprocity”: 4 ≥ 4), and therefore he was regarded as an individual with ASD. All of the eight participants who underwent the DISCO and ADOS satisfied the criteria for ASD for both measures. All participants provided written informed consent. This study was conducted in accordance with the principles of the Declaration of Helsinki and was approved by the Ethics Committee of the Faculty of Medicine of Showa University.

### Data acquisition

All scans were acquired using a 1.5T GE Signa system (General Electric, Milwaukee, WI, USA). The resting-state functional images were acquired using a gradient echo-planar imaging sequence (in-plane resolution: 3.4375 × 3.4375 mm, echo time (TE): 40 ms, repetition time (TR): 2000 ms, flip angle: 90°, slice thickness: 4 mm with a 1-mm slice gap, matrix size: 64 × 64, 27 axial slices). Two-hundred and eight volumes were acquired in a single run, and the first four volumes were discarded to allow for T1 equilibration. We also obtained a high-resolution T1-weighted spoiled gradient recalled 3D MRI image (in-plane resolution: 0.9375 × 0.9375 mm, 1.4 mm slice thickness, TR: 25 ms, TE: 9.2 ms, matrix size: 256 × 256, 128 sagittal slices). Each participant was instructed to lie still with his eyes closed and to not think of anything in particular, yet stay awake in the dim scanner room.

### Data preprocessing

With the exception of the pinpoint use of Analysis of Functional NeuroImages (AFNI) [26], we mainly used the Statistical Parametric Mapping software (SPM 8) (Wellcome Department of Cognitive Neurology, London, UK) to preprocess the resting-state functional magnetic resonance imaging (rs-fMRI) data. Unless otherwise specified, rs-fMRI data were preprocessed using functions implemented in the SPM software. First, functional images were adjusted for slice timing and then were corrected for head motion. None of the participants moved translationally more than 2 mm (*x*, *y*, and *z*) or rotated more than 2° (roll, pitch, and yaw) in relation to the first volume. We next skull-stripped the functional images using 3dAutomask and 3dcalc and despiked the time series using 3dDespike in AFNI to account for the impact of outliers. The functional images were then co-registered to each participant’s T1 image and normalized to the Montreal Neurological Institute (MNI) template, resampled to a resolution of 2 × 2 × 2 mm. Finally, the images were smoothed using a 6-mm Gaussian kernel.

We calculated covariates from the segmented white matter and cerebrospinal fluid using the “aCompCor” method [27] in order to remove artefactual components from the fMRI time series. These covariates, together with 12 head motion parameters (6 motion parameters and their first-order temporal derivatives), were then regressed out from the smoothed rs-fMRI data. To further remove possible effects of sub-millimeter motion on estimations of functional connectivity, we applied the scrubbing method in the following manner [28, 29]: (1) the framewise displacement (FD) and frame-by-frame signal intensity change (DVARS) indices were calculated immediately after head motion correction; (2) we regarded a volume as a motion-contaminated volume either when its FD value was greater than 0.5 mm or when its DVARS value was greater than the 75th percentile plus 1.5 times the interquartile range; (3) the signal values of the motion-contaminated volumes were interpolated by applying the cubic spline function [28]; (4) a band-pass filter (0.009–0.08 Hz) was applied to further reduce the effects of low-frequency drifts and high-frequency physiological noise; (5) finally, the interpolated volumes were deleted. There were no significant differences between the two groups either in the mean (± standard deviation [SD]) FD (0.115 ± 0.042 in TD, 0.107 ± 0.049 in ASD; *p* = 0.495) or in the mean (± SD) DVARS (TD: 12.94 ± 2.05, ASD: 12.86 ± 1.83, *p* = 0.863). The number of deleted volumes after the scrubbing procedures was 5.24 ± 4.90 for the TD group and 4.25 ± 4.23 for the ASD group. No significance difference was found between groups (*p* = 0.36).

### Connectivity-based functional parcellation

**Fig. 1:**
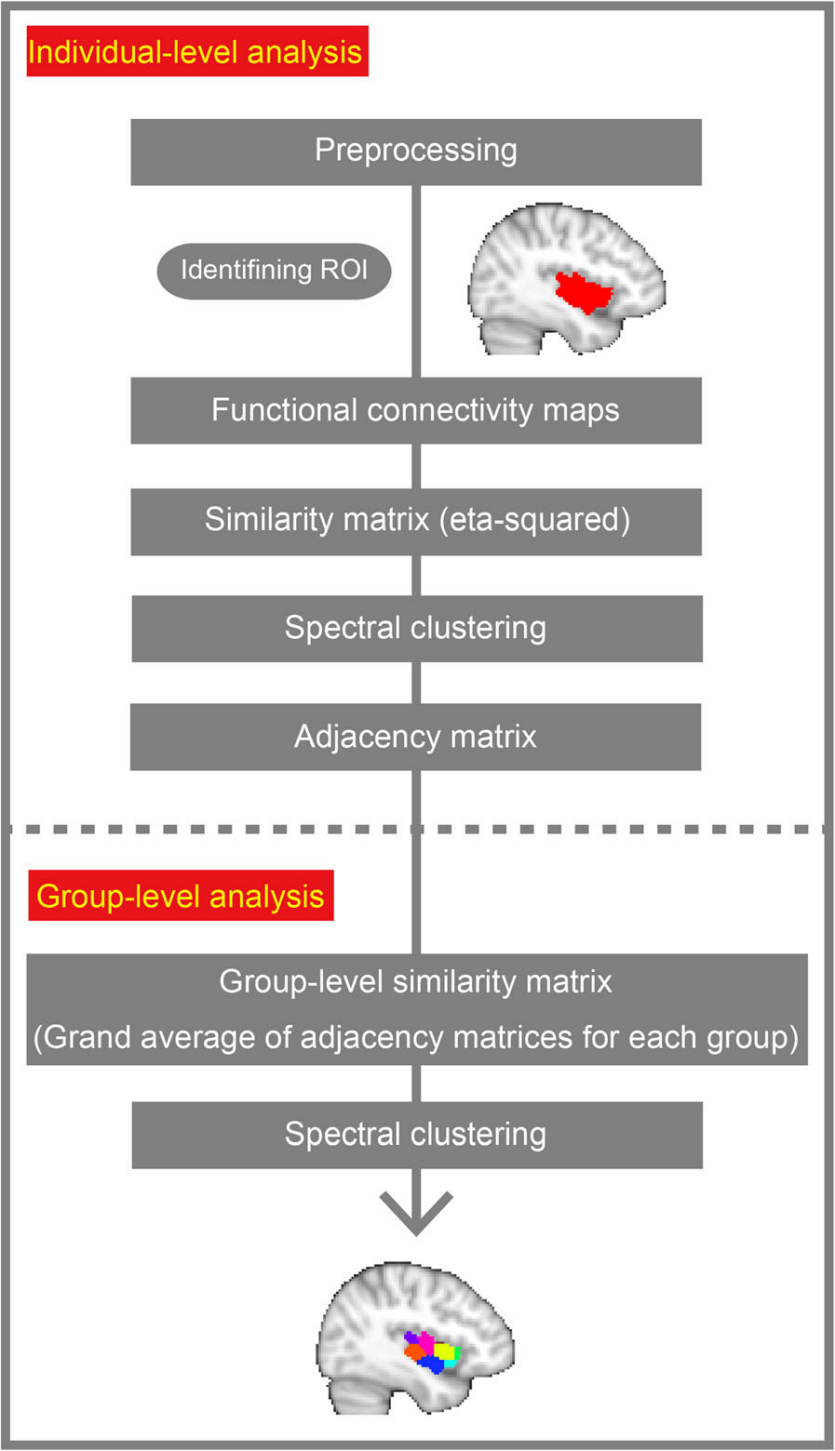
The procedure for the functional connectivity-based parcellation of the insula. We first identified voxels in the left and right insula of each participant and generated a set of functional connectivity maps by correlating the resting-state time-series of each voxel with voxels in a whole gray matter mask (excluding the insula) for each hemisphere. Following a Fisher’s z-transformation for functional connectivity maps, we constructed the individual-level similarity matrix using eta-squared, which is a measure of similarity between a pair of functional connectivity maps (see the “Connectivity-based functional parcellation” section). We applied the spectral clustering algorithm to the set of individual-level similarity matrices in order to cluster voxels with similar time-series of the resting-state signal fluctuations. For group-level analysis, we first calculated a binary adjacency matrix for each participant. Adjacency matrices of all participants were averaged separately for TD and ASD individuals to generate a group-level similarity matrix. Finally, we applied the spectral clustering algorithm to the group-level similarity matrix to assign a *k* clustering label to each voxel (see the “Connectivity-based functional parcellation” section)

Figure 1 illustrates the processing scheme for the connectivity-based parcellation of the insula. Briefly, we generated a group of functional connectivity maps for each participant using every voxel in the insula as a seed. We then applied a clustering method to the resulting groups of connectivity maps for the left and the right insula separately and identified a set of voxel clusters such that each voxel in the same cluster had a similar connectivity pattern. After calculating the cluster map for each individual, we again applied the clustering method to matrices that represent patterns of similarity of clustering either within the TD or the ASD group to obtain separate group-level functional parcellation maps for the TD and ASD groups.

Specifically, we first identified all of the voxels included in the insula as follows: (1) voxels in the standard spaces of the left and the right insula were defined using the Harvard-Oxford probabilistic atlas thresholded at 25 % probability; (2) voxels in the insula from both hemispheres were further constrained by conjunction with a study-specific GM mask, which represented voxels that survived the threshold of 40 % GM probability in every participant. This method of generating the study-specific GM masks was based on previous studies [30, 31]. This procedure resulted in the inclusion of 1161 voxels in the left insula and 1179 voxels in the right insula.

Next, we generated a group of voxel-based functional connectivity maps by calculating temporal correlations between time series of each individual voxel in the insula and those of all voxels within the study-specific GM mask, except for the voxels in the insula. This procedure resulted in the generation of 1161 maps for the left insula and 1179 maps for the right insula in each participant. Connectivity maps of the Pearson’s correlation coefficients were then converted to *Z*-scores using the Fisher’s *r*-to-*z* transformation.

For the functional parcellation, we evaluated the similarities of functional connectivity patterns among the 1161 maps for the left insula and the 1179 maps for the right insula. Following previously described methods [19, 32–34], we calculated the eta-squared value as a measure of similarity between a pair of connectivity maps as follows:

$$ \mathrm{eta}\ \mathrm{squared} = 1\ \hbox{--}\ \frac{{\displaystyle {\sum}_{i=1}^n}\left\{{\left({a}_i-{m}_i\right)}^2+{\left({b}_i-{m}_i\right)}^2\right\}}{{\displaystyle {\sum}_{i=1}^n}\left\{{\left({a}_i-X\right)}^2+{\left({b}_i-X\right)}^2\right\}} $$

where *a*_*i*_ and *b*_*i*_ are the *Z*-scored connectivity values at voxel *i* in connectivity maps *a* and *b*, respectively. *m*_*i*_ is the mean of the two connectivity map values at voxel *i*, and *X* is the mean of all voxels of the two connectivity maps. Using the eta-squared values between all pairs of connectivity maps, we constructed the individual-level similarity matrix for the left and the right insula separately (1161 × 1161 matrix for the left insula and 1179 × 1179 matrix for the right insula). After determining the optimal number of clusters (*k*, see the “Estimation of optimal number of clusters” section), we applied the spectral clustering algorithm to each of the individual-level similarity matrices and parcellated either the left or the right insula into *k* clusters based on similarities in functional connectivity patterns.

After performing functional parcellation at the individual-level, we next performed group-level functional parcellation as follows [33]: (1) we generated a binary adjacency matrix whose value was 1 if a pair of voxels belonged to the same cluster and zero otherwise for each participant, (2) we then generated a group-level similarity matrix by averaging the adjacency matrices of all individuals within each group, and (3) we applied the spectral clustering algorithm to the group-level similarity matrix to assign one of the *k* clustering labels to each voxel.

The spectral clustering algorithm arbitrarily assigns one of the *k* clustering labels to each voxel. To compare the parcellation results of the two groups, we first fixed the configuration of labels in the TD group as a reference. Then, for each of the possible instantiation of the *k* labels in the ASD group, we calculated the ratio of the voxels having the same label to those having different labels in the two groups. We selected the configuration of labels that maximized this ratio.

### Estimation of the optimal number of clusters

Before applying the spectral clustering method, we determined the optimal number of clusters (*k*) using two complementary indices that evaluate the goodness of clustering solutions: variation of information (VI) and mutual information (MI) [35]. VI is an information-theoretic measure that quantifies the information lost and gained in changing clustering solution A to clustering solution B. This measure can thus be used as an index of dissimilarity [19]. On the other hand, MI is an information-theoretic measure that represents the similarity between clustering solutions. We examined the VI and MI values from 2 to 10 for each *k* value for the left and the right insula and for the TD and ASD groups separately and selected the optimal *k* value. A *k* value with a low VI and a high MI indicates a good solution in terms of similarity.

To calculate VI and MI, we used a split-half procedure, as described previously [36, 37]. First, in order to determine an optimal *k* value not biased toward the TD or ASD groups, all of the participants were randomly assigned to one of two groups (group A and group B). We then applied the aforementioned spectral clustering algorithm to each group for each *k* (*k* = 2, 3,…, 10) and obtained group-level parcellation maps for each group. Finally, we compared the clustering of the two groups by calculating VI and MI for each *k*. We repeated this procedure 100 times, as in previous studies. A previous functional connectivity-based parcellation study on the insula demonstrated that the stability of the solutions substantially decreases when the number of clusters (*k*) exceeds 10 [19]. Therefore, we explored *k* values ranging from 2 to 10 in the present study.

In order to determine the optimal *k* value, we first identified a set of “good solutions” using VI and MI independently and then determined the optimal values using the logical product of the two solutions. Since VI is a measure of dissimilarity, we identified points (*k* values) of “local minimal” VI as good solutions. Similarly, since MI is a measure of similarity, we identified points (*k* values) of “local maximal” MI as good solutions. Here, a local minimum of VI is defined as a range of points with a significantly smaller value than its two adjacent points. The local maximum MI value was determined in a similar manner. We determined the optimal *k* value as the point where local minimal VI and local maximal MI values converge.

We also performed another split-half procedure within the TD group by randomly assigning participants with TD into one of two groups (group 1 or group 2) while changing *k* from 2 to 10. We did this to ensure that the optimal *k* value determined by our procedure was not biased due to contamination from the ASD group.

### Comparison of functional parcellation patterns between the TD and ASD groups

After the parcellation of the insula in each participant, we examined between-group differences in the volumes of specific sub-regions by performing a permutation test, as previously described [33]. Briefly, we randomly assigned participants to one of the two groups by permuting the diagnostic labels of all of the participants and then applied our clustering algorithm to each of the permuted groups. The sub-regions in each group were re-labeled following the aforementioned procedure, which used the labels from the original TD group as references (see the “Connectivity-based functional parcellation” section). This procedure was repeated 5000 times to obtain the null distribution of volumetric differences between the groups. We deemed between-group volumetric differences statistically significant when the difference between the TD and ASD groups fell above the 95th percentile of the null distribution.

### Meta-analytic decoding of the sub-regions’ connectivity maps

In addition to analyzing sub-regional volumes, we also examined whether there are significant alterations in functional characteristics of insular sub-regions in ASD. We thus examined the functional profiles of each sub-region in the TD and ASD groups separately using a recently developed meta-analytic tool called “Neurosynth” [18]. Given a pattern of functional connectivity as an input, the tool allows us to decode the relevant psychological and physiological functions (e.g., “cognitive control,” “emotion,” and “perception”) strongly associated with that pattern.

As a first step, we performed functional connectivity analysis using each of the insular sub-regions as a region of interest (ROI). We generated maps of the Pearson’s correlation coefficients between the averaged time series of a sub-region and those of all voxels within the GM mask for each individual. After transformation into *z*-maps, the maps of all participants in the TD and ASD groups were separately subjected to a one-sample *t* test using age as a nuisance covariate to obtain the connectivity pattern at the group level. The resulting *t-*statistic maps were converted to *z*-statistic maps and then fed into Neurosynth as inputs for meta-analytic decoding.

In order to characterize the functional properties of each functional connectivity map, we determined the top 5 terms that showed the strongest correlations with each functional connectivity map. Related terms (e.g., “emotion” and “emotional”) were merged into a single term of the base form. This procedure yielded a total of 14 terms, as some of the same terms were repeatedly selected across all sub-regions for both hemispheres. Finally, we drew a radar chart consisting of these 14 terms to visually assess the potential psychological and physiological functions of each sub-region as functional fingerprints. To visualize the similarities (and dissimilarities) of these functional fingerprints, we constructed a 14-dimensional feature vector whose elements were the correlation coefficients of the 14 terms for each sub-region. We then calculated the Pearson’s correlation coefficient between pairs of feature vectors to represent the similarities between sub-regions in terms of functional fingerprints.

### Connectivity-based parcellation of the intracalcalrine cortex as a control region

The functional parcellations of the insula resulted in different patterns between the TD and ASD groups (see the “Visual inspection of functional connectivity-based parcellation” section). To examine whether group differences in the parcellation patterns have any regional selectivity, we applied the same clustering analysis to the intracalcarine cortex as a control region.

The intracalcarine cortex as defined in the Harvard-Oxford probabilistic atlas (thresholded at 25 probability) roughly corresponds to the primary visual cortex and its neighboring regions. Given the evidence of alterations in vision-related functions in some ASD individuals (e.g., enhanced visual search ability [38, 39]), it may not be possible to strongly assert that functional organizations within the lower-order visual cortices are unaltered in the ASD brain. However, we propose that the intracalcarine cortex may serve as a control for the insula for the following reasons: (1) the region is thought to be dedicated to visual processing (in contrast to the multiple distinct functions of the insula) and (2) the size of the region (1405 voxels) is comparable to those of the left and right insula (left: 1161 voxels, right: 1179 voxels).

We estimated the optimal cluster number in the intracalcarine cortex using MI and VI in the same manner as that for the left and right insula (see the “Estimation of the optimal number of clusters” section). After determining the optimal number of clusters of this region, the parcellation patterns were visually inspected and compared between groups. Furthermore, a possible volumetric alteration was examined using a permutation test for each sub-region (see the “Comparison of functional parcellation patterns between the TD and ASD groups” section).

### Assessment for the replicability of functional parcellation patterns

To assess the stability of the functional parcellation patterns, we performed a replication analysis using subsets of the data. First, we divided the participants of each group into six folds (in the case of the ASD group, 6 participants per fold [36 participants in total]). The stability of the parcellation pattern was examined using the leave-one-fold-out method. In other words, we applied the clustering method to the data for the remaining five folds and repeated the procedure for six times until all six folds were excluded. This procedure resulted in six parcellation patterns. We then examined the similarity between the parcellation pattern using all participants in the group and each of the six parcellation patterns that were generated using the leave-one-fold-out method. We used the MI as an index of similarity of the two patterns.

## Results

### Optimal number of clusters

**Fig. 2:**
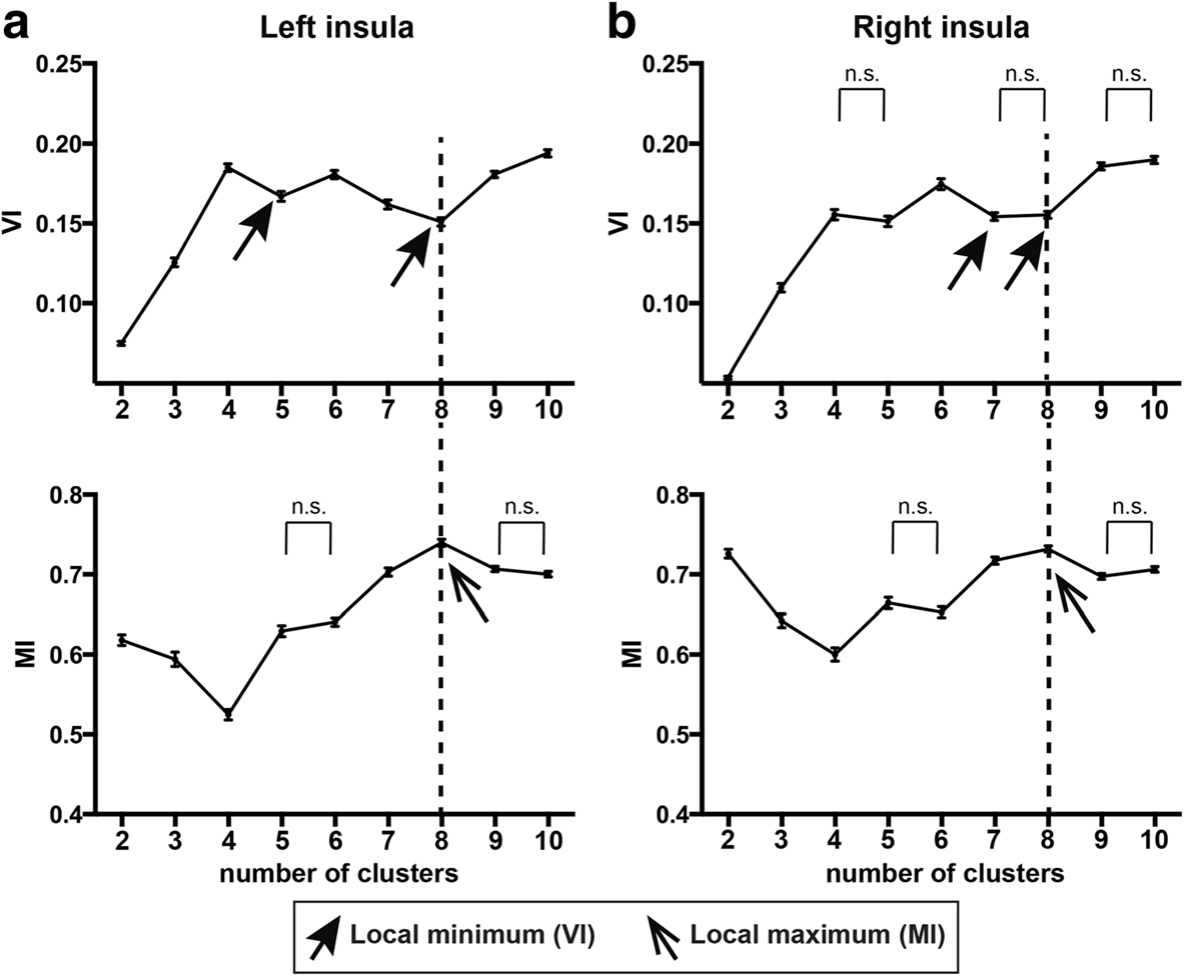
Determination of the optimal number of clusters based on VI and MI. The VI and MI values are shown for every clustering solution for *k* values ranging from 2 to 10 for each side of the insula (**a** Left insula, **b** Right insula). *Arrows* indicate either local minima of VI or local maxima of MI. *Dashed lines* denote the optimal number of solutions as determined using both VI and MI. The *error bars* denote standard errors of the mean for 100 repetitions of the split-half procedure (see the “Estimation of the optimal number of clusters” section). *n.s.* indicates no statistically significant difference between points

Figure 2 shows the means and standard errors of the mean (SEM) of VI and MI for the left and the right insula for each clustering solution. In the left insula, VI had local minima at *k* = 5 and 8 (VI: 0.167 ± 0.0031 [mean ± SEM] for *k* = 5 and VI: 0.151 ± 0.0026 for *k* = 8), while MI had a local maximum at *k* = 8 (MI: 0.740 ± 0.0046) (Fig. 2a). Following the criteria for the selection of the optimal number of clusters using VI and MI (see the “Estimation of optimal number of clusters” section), we determined an optimal *k* of 8 for the left insula. In the right insula, VI had local minima at *k* = 7 and 8 (VI: 0.154 ± 0.0024 for *k* = 7 and VI: 0.155 ± 0.0022 for *k* = 8), while MI had a local maximum at *k* = 8 (MI: 0.732 ± 0.0039) (Fig. 2b). We determined an optimal *k* of 8 for the right insula using the information that we obtained from VI and MI.

### Visual inspection of functional connectivity-based parcellation

**Fig. 3:**
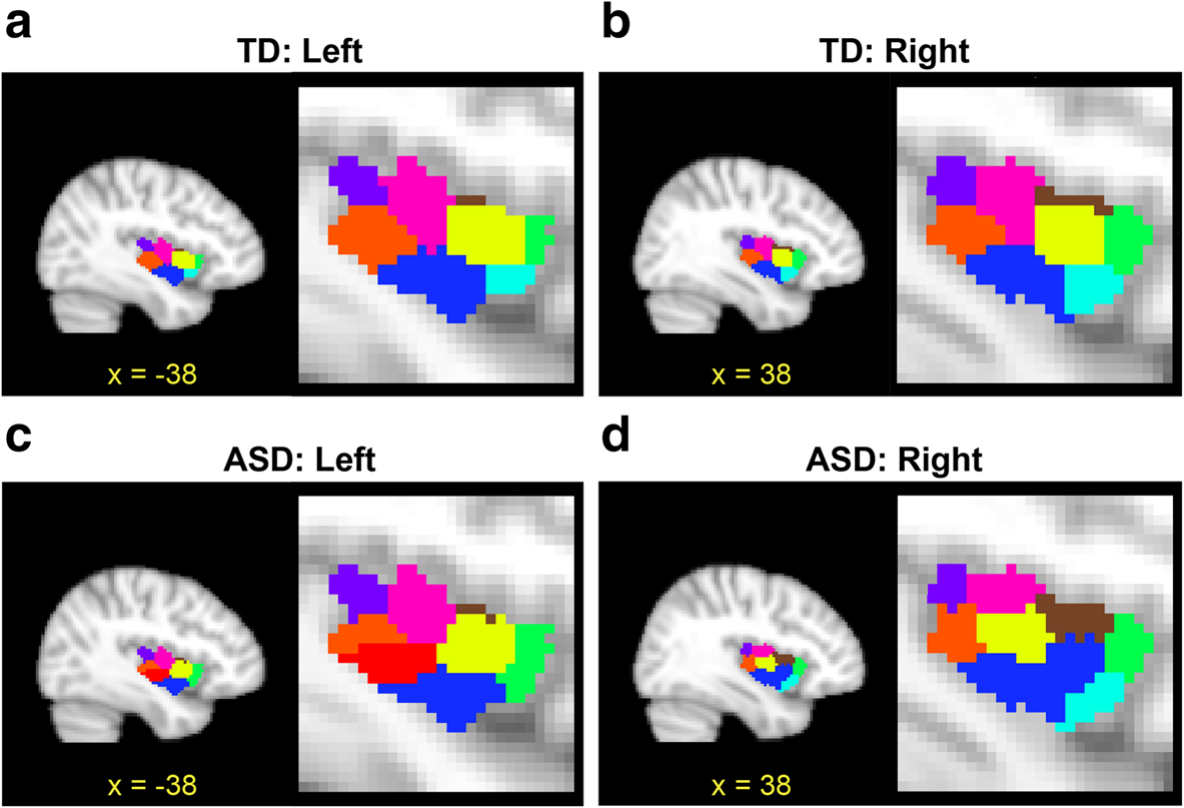
The patterns of functional parcellation in the left and right insula in TD and ASD (**a** Left insula of TD, **b** Right insula of TD, **c** Left insula of ASD, **d** Right insula of ASD). Each figure is presented in sagittal and magnified sagittal views. The color of each insular sub-region reflects the color of the corresponding sub-region in the TD and ASD groups

**Fig. 4:**
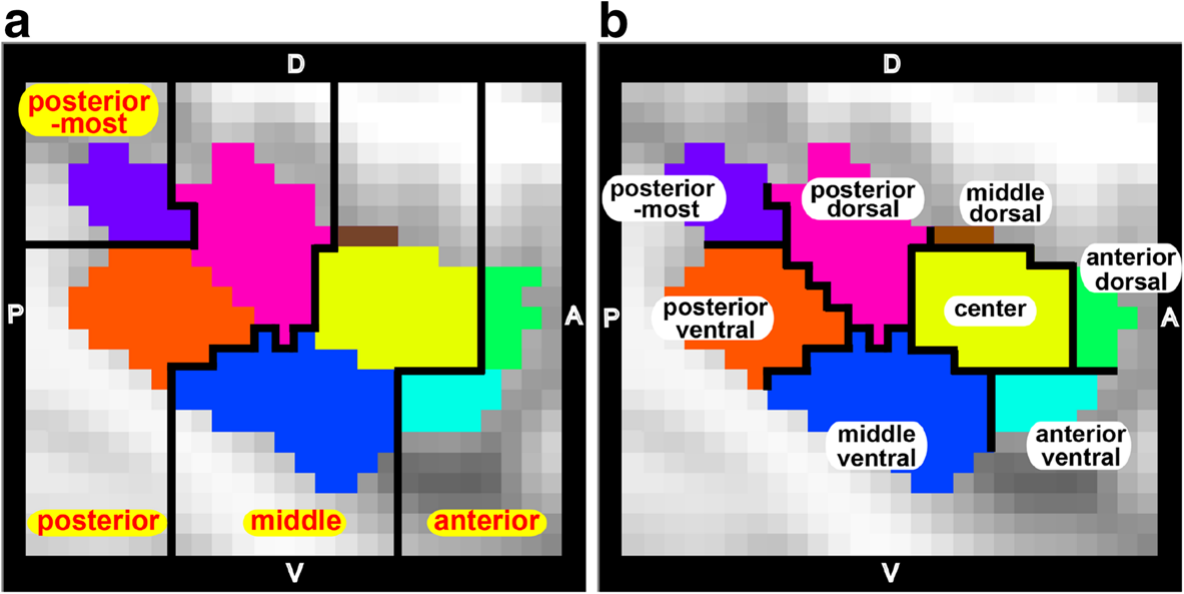
Spatial configurations and labels for the insular sub-regions. The parcellation pattern of the left insula of the TD group is magnified. We divided the whole region anterior, middle, posterior, and posterior-most sectors along the anterior-posterior axis (**a**). Further subdivision along the dorsal-ventral axis in each sector resulted in eight sub-regions in total (**b**): (1) anterior sector: anterior dorsal (AD) and ventral (AV) sub-regions (*green* and *cyan*), (2) middle sector: middle dorsal (MD), central (C), and middle ventral (MV) sub-regions (*brown*, *yellow*, and *blue*), (3) posterior sector: posterior dorsal (PD) and ventral (PV) sub-regions (*magenta* and *orange*), and (4) posterior-most sub-region (*purple*). *D* dorsal, *V* ventral, *A* anterior, *P* posterior

Figure 3 illustrates the functional parcellation patterns of the left and right insula in the TD and ASD groups when *k* = 8. We named the eight sub-regions based on their locations along the anterior-posterior and dorsal-ventral axes. Figure 4 shows the magnified picture of the parcellation pattern of the left insula of the TD group (Fig. 3a). We first divided the whole region anterior, middle, posterior, and posterior-most sectors along the anterior-posterior axis (Fig. 4a). Each of the anterior and posterior sectors was further divided into dorsal and ventral sub-regions. The 3 sub-regions in the middle sector were labeled as middle dorsal, center, and middle ventral sub-regions (Fig. 4b). We thus obtained eight sub-regions as follows: (1) anterior sector: anterior dorsal (AD) and ventral (AV) sub-regions; (2) middle sector: middle dorsal (MD), central (C), and middle ventral (MV) sub-regions; (3) posterior sector: posterior dorsal (PD) and ventral (PV) sub-regions; and (4) posterior-most sub-region. Note that this labeling is similarly applicable to the parcellation pattern in the right insula (Fig. 3b). We also note that our parcellation patterns were highly similar to previously identified patterns based on resting-state FC studies of a neurotypical population [19]. Most notably, the parcellation patterns in our study and those in the previous study were consistent in that the anterior and posterior sectors were divided into two sub-regions and the middle sector was divided into three sub-regions.

In comparison to the parcellation patterns of the TD group, we observed notable localized alterations in both the left and the right insula in the ASD group. Specifically, we observed that the left anterior sector, which was divided into two sub-regions in the TD group, contained only a single sub-region in the ASD group (Fig. 3c). Moreover, the left PV sub-region, which was a single region in the TD group, was divided into two sub-regions in the ASD group. On the other hand, the parcellation patterns of the middle and posterior-most sectors in the ASD group were largely unchanged from those of the TD group. In the right insula, the MV sub-region was expanded and the central sub-region was shifted in the posterior direction in the ASD group (Fig. 3d). Aside from localized alterations, the parcellation patterns in the anterior and posterior-most sectors were largely the same in the two groups. These observations indicate that the organization of functional sub-regions may be altered in specific parts of the left and right insula in ASD.

### Meta-analytical decoding of the left insular sub-regions

**Fig. 5:**
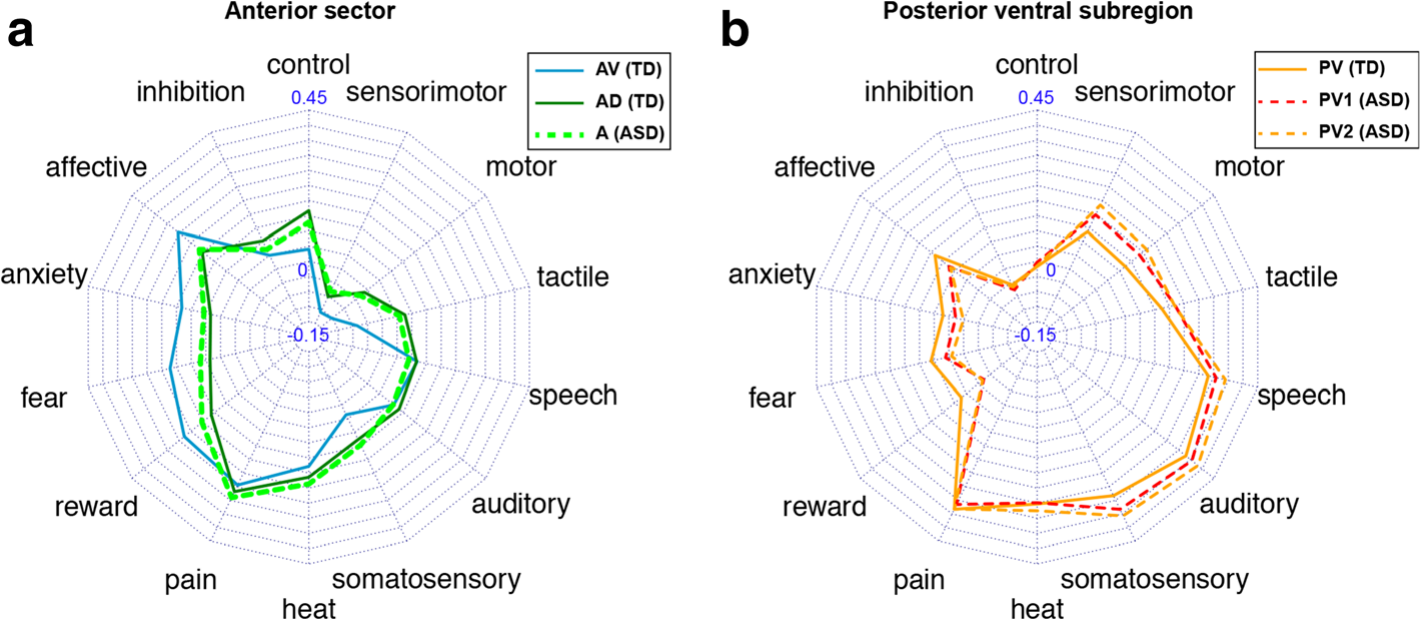
The meta-analytic decoding of sub-regions in the left anterior sector and posterior ventral sub-regions. **a** The radar chart that shows the correlation of left anterior ventral (AV) and anterior dorsal (AD) sub-regions in the TD group and the anterior section in the ASD group with the 14 terms of interest. Note that the profile of the ASD anterior sector is more similar to that of the AD sub-region rather than the AV sub-region in the TD group. **b** The radar chart that shows the correlation of left posterior ventral (PV) sub-region in the TD group and the two parcels within the PV sub-region (PV1 and PV2) with the 14 terms of interest. The profiles of these three sub-regions were highly similar

We investigated the functional profiles of the anterior sub-regions and the PV sub-region in the left insula, where the functional parcellations were visually different between the TD and ASD groups. First, we performed a meta-analytic decoding of the functions of the two anterior sub-regions (AD and AV) in the TD group and of the anterior sub-region in the ASD group. The results are presented as a radar chart in Fig. 5. The functional profile of the anterior sub-region in the ASD group showed an almost identical pattern to that of the AD sub-region in the TD group (*r* = 0.97). Although the profile of the ASD anterior sub-region also significantly correlated with that of the AV sub-region in the TD group (*r* = 0.86), the profile of the anterior sub-region in the ASD group was clearly more similar to that of the AD sub-region in the TD group. Therefore, the alterations of the anterior sector in the ASD group may be characterized as the absence of the AV sub-region together with the volumetric expansion of the AD sub-region. The volumetric increase was statistically confirmed by the permutation test (ASD anterior sub-region > TD AD sub-region, *p* = 0.024) (Additional file 1: Figure S1a). When the functional profiles were compared between the AD and AV sub-regions, the AV sub-region was more highly associated with terms related to affection and emotion, such as “reward,” “fear,” “anxiety,” and “affective,” whereas the AD sub-region was more preferably associated with a cognitive terms (“control” and “inhibition”) and sensorimotor terms (“sensorimotor,” “motor,” “tactile,” and “somatosensory”).

We next performed a meta-analytic decoding of the function of the PV sub-region in the TD group and functions of the two PV sub-regions in the ASD group. For simplicity, we will label the anterior portion of the PV sub-region in the ASD group as PV1 and the posterior PV as PV2. When comparing the PV sub-region in the TD group with the two PV sub-regions in the ASD group, we observed almost identical functional profiles among these sub-regions (TD PV and ASD PV1: *r* = 0.96; TD PV and ASD PV2: *r* = 0.95; ASD PV1 and ASD PV2: *r* = 1.00).

### Meta-analytical decoding of the right insular sub-regions

**Fig. 6:**
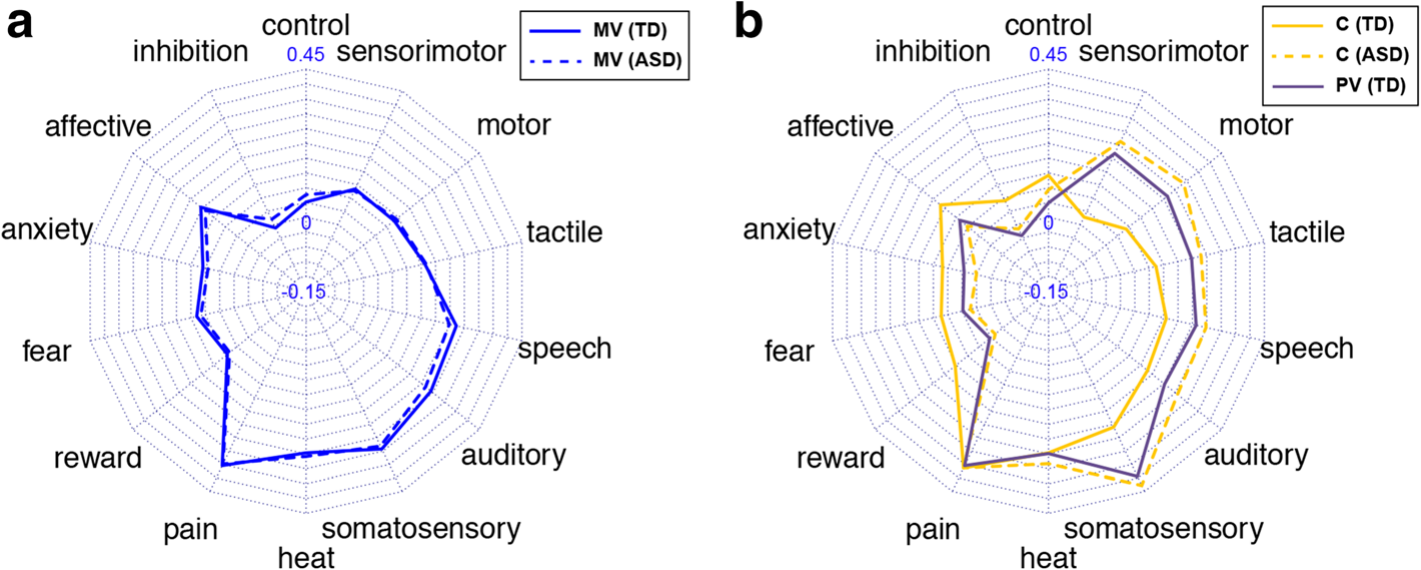
The meta-analytic decoding in the right middle ventral, central, and posterior ventral sub-regions. **a** The radar chart that shows the correlation of the middle ventral (MV) sub-region in both groups with the 14 terms of interest. **b** The radar chart shows the correlation of the central (C) and posterior ventral (PV) sub-regions in both groups with the 14 terms of interest

We next examined functional alterations in the right insular sub-regions of the ASD group. We previously noted the visual observation suggesting the extension of the MV sub-region into the more dorsal part of the insula (see the “Visual inspection of functional connectivity-based parcellation” section). We statistically confirmed this observation by using the permutation test and finding a significant group difference (ASD > TD, *p* = 0.003) (Additional file 1: Figure S1b). Meta-analytic decoding of the MV sub-region revealed that the region was strongly associated with terms related to perceptual processes such as “pain,” “somatosensory,” “auditory,” “speech,” and “heat,” and that such functional profiles were almost identical between the TD and ASD groups (*r* = 0.99) (Fig. 6a). In addition to the spatial expansion of the MV sub-region, our visual inspection indicated that the position of the C sub-region had shifted in the posterior direction in the ASD group. In order to examine possible functional alterations in this region, we performed meta-analytic decoding of the C sub-region in the two groups and found highly dissimilar patterns (*r* = 0.45). However, we observed that the C sub-region in the ASD group was functionally similar to the PV sub-region in the TD group (*r* = 0.98), which was strongly associated with terms largely related to perceptual (“somatosensory,” “tactile,” and “auditory”) and motor processes. The results are consistent with the observation of the spatial shift of the C sub-region toward the posterior direction in the ASD group.

### Group differences in individual variability in insula parcellation

Our analysis showed significant differences in functional parcellation patterns between the TD and ASD groups. However, recent studies have suggested that the ASD brain is highly idiosyncratic, indicating that each individual ASD brain may be greatly discrepant from the group mean [40]. Therefore, it may be argued that the inter-individual variability in the insula parcellation patterns is larger in the ASD than in the TD and that such enhanced variability might have played a role in the altered parcellation pattern in the ASD. To test this possibility, we calculated the MIs between each individual adjacency matrix of the ASD participants and the group-level similarity matrix (see the “Connectivity-based functional parcellation” section) of the ASD group when the cluster number (*k*) was optimally set to 8 (eight sub-regions). Similarly, the MIs were also calculated for the TD individuals using the TD group-level similarity matrix. If the parcellation pattern in each individual brain is more idiosyncratic in the ASD group, then the MI should be significantly reduced in the ASD group than in the TD group. However, we did not observe a significant difference in MIs between the ASD and TD groups in either the left or right insula (left insula: *t* = −1.54, *p* = 0.13, right insula: *t* = 0.038, *p* = 0.97).

We also reasoned that the degree of idiosyncrasy in the insular parcellation pattern of an individual might be associated with his or her severity of the ASD symptoms. To explore this possibility, we first evaluated the degree of idiosyncrasy using the MI values between the adjacency matrix of each ASD individual and the group-level similarity matrix of ASD individuals. These MIs represented the discrepancy from the mean ASD parcellation pattern. Then we computed the correlation between the AQ scores and the MI values, but the correlation was not significant (left insula: *r* = 0.03, *p* = 0.85, right insula: *r* = −0.02, *p* = 0.90). We also calculated the individual MIs using the group-level similarity matrix of TD individuals, as indices for the discrepancy from the typical (mean TD) parcellation pattern. However, no significant correlation with the total AQ score was found in either the left or right insula (left insula: *r* = 0.04, *p* = 0.83, right insula: *r* = −0.02, *p* = 0.90).

### Functional parcellation in the intracalcarine cortex

The same clustering methods as those used for the insula were applied to the control region of the intracalcarine cortex. MI reached the local maximum at *k* = 7, 8, 9, and 10, and VI reached the local minimum at *k* = 6 and 7 (Additional file 2: Figure S2). Following the same criteria as that for the insula, we determined an optimal *k* of 7. Based on visual inspection, the functional parcellation patterns at *k* = 7 were highly comparable between the TD and ASD groups (Additional file 3: Figure S3). Permutation tests for the volume of each sub-region revealed no significant group differences in any of the seven sub-regions (light blue region: *p* = 0.36, green region: *p* = 0.67, orange region: *p* = 0.55, yellow region: *p* = 0.58, magenta region: *p* = 0.55, blue region: *p* = 0.52, violet region: *p* = 0.31). The comparable parcellation pattern between groups in the control region indicates that group differences that were identified by our connectivity-based parcellation may be selective to parts of brain regions, including the left and right insula.

### Replicability of the functional parcellation patterns in the insula

We examined the replicability of the functional parcellation patterns using the leave-one-fold-out method (six folds in each group) (see the “Assessment for the replicability of functional parcellation patterns” section). The means and SEM of MIs between the parcellation pattern for all participants in a group and each of the six parcellation patterns that were generated using the leave-one-fold-out method were as follows: TD: 0.88 ± 0.02 (mean ± SEM), ASD: 0.81 ± 0.01 for the left insula; TD: 0.90 ± 0.02, ASD: 0.79 ± 0.01 for the right insula. These high MI values indicate good stability for the parcellation patterns of the left and right insula in both groups.

We also examined the replicability of the group differences in the sub-regional volumes that were identified in the left and right insula. In the left insula, the anterior sub-region of the ASD group was significantly larger than the AD sub-region of the TD group (see the “Meta-analytical decoding of the left insular sub-regions” section). In the right insula, the MV sub-region was significantly larger for the ASD group than the TD group (see the “Meta-analytical decoding of the right insular sub-regions” section). For these two sub-regions, we subtracted the volume of the ASD group from that of the TD group for each of the six leave-one-fold-out steps. Because the aim of this analysis was to test whether the subtracted value is significantly lower than 0, we performed a one-sample, one-tailed *t* test using the six samples. As a result, we observed a significant difference for both sub-regions (the left sub-region: *t* = −2.88, *p* = 0.017; the right sub-region: *t* = −2.64, *p* = 0.023). Therefore, the group differences in the sub-regional volumes were also replicable.

## Discussion

In this study, we performed an automatic functional parcellation of the insular cortex in adult male ASD by adopting a data-driven clustering method based on resting-state FC patterns of voxels. In the comparison with matched TD brain, we observed notable alterations in specific sub-regions in the left and right insular cortices of ASD brain. These alterations were localized to the anterior sub-regions of the left insula and the middle ventral sub-region of the right insula. In the left insula, whereas the anterior sector contained two functionally differentiated sub-regions in TD brain, only a single functional cluster was identified in the anterior sector in the ASD group. No clear group-difference was observed in the control region of the intracalcarine cortex using the same resting-state FC-based parcellation method. Additional analyses using subsets of the entire dataset confirmed the high stability of the insular parcellation patterns in both groups. Meta-analytical decoding revealed the absence of sub-region specialized for emotional and affective functions in the anterior sector in the ASD brain. The middle ventral sub-region of the right insula, which is primarily specialized for sensory and auditory-related functions, extended into the more dorsal parts of the insula and its volume was significantly enlarged compared with the same region in the TD brain. We thus observe alterations in the functional organization of the left and right insular sub-regions, which may underlie abnormalities in emotional/affective and sensory domains in ASD.

### The left insula lacks an emotion-related sector in ASD

We found a notable difference in the anterior sector of the left insula when we carried out a group comparison of functional parcellation patterns. Specifically, whereas the anterior sector was parceled into dorsal and ventral sub-regions in the TD group, only a single cluster was identified in the anterior sector in the ASD group. Analysis of the functional profiles of the two sub-regions indicate a functional differentiation in the TD brain, such that the ventral sub-region is characterized by selective involvement in emotional and affective processes, fear, anxiety, and reward, whereas the dorsal sub-region is more strongly involved in cognitive and sensorimotor functions compared with the ventral sub-region. On the other hand, the functional profile of the single anterior cluster in the ASD brain closely matched that of the dorsal rather than the ventral sub-region in the TD brain. Therefore, we report that the left anterior insula in ASD is functionally altered and is characterized by the absence of the sub-region for emotional and affective functions.

It is notable that such an alteration in functional parcellation was observed in the left insula. Based on anatomical evidence of left-to-right asymmetry in peripheral autonomic efferent neurons and homeostatic afferent neurons, as well as a review of neuroimaging literature, an influential model of the insula posits that the left insula is associated with parasympathetic (“affiliative”) functions, while the right side is associated with sympathetic (“aroused”) functions. This idea provides the neurobiological foundation for the functional laterality of emotion. According to this model, the left anterior insula is more strongly involved in “positive (energy enrichment)” emotions. Consistent with this view, previous fMRI studies of neurotypical populations demonstrated the left-lateralized activation of the anterior insula during the viewing of pleased facial expressions [41] and when mothers viewed their own child [42] and experienced maternal and romantic love [43]. These observations indicate a dichotomy of emotion, so that the left anterior insula may be viewed as critical for group-oriented (affiliative) emotions as opposed to individual-oriented emotions.

Our observation of the absence of the anterior sub-region for emotional and affective functions in ASD may predict abnormalities in group-oriented emotions. Consistently, a recent model of ASD focused on the concept of “social motivation” and proposed that the core problems of ASD may be explained by extremely diminished social motivation [44]. According to previous surveys, half of the adults with ASD report having no particular friends [45] and score significantly lower on items in the friendship questionnaire that concern attitudes toward interpersonal relationships, such as pleasure in close friendship and interest in people [46]. Therefore, the functional alteration in the anterior section of the left anterior insula may provide a neurological basis for the lack of group-oriented emotion and behavior in ASD.

### The right insula has an enlarged sensory and auditory-related section in ASD

We identified a significant alteration of functional parcellation patterns in the right insula. Specifically, we observed an increase in the volume of the middle ventral sub-region, which is specialized for auditory-related and other sensory functions. Convergent evidence from anatomical, electrophysiological, brain lesion, and neuroimaging studies indicate that the insular cortex is involved in multimodal sensory functions, including somatosensory, olfactory, visceral, and auditory functions [47]. In particular, previous reports have highlighted a critical role for the insula in various aspects of auditory function, including auditory attention, music, the processing of novel auditory stimuli, temporal processing, and visual-auditory integration [47]. Our analyses of the functional profiles of insular sub-regions indicate that the middle ventral sub-region is specifically associated with such auditory and sensory functions.

Given the functional profiles of the above insular sub-regions, it is plausible that the volumetric expansion of the right middle ventral sub-region is associated with sensory abnormalities in ASD, including those in the auditory domain. Indeed, atypical sensory reactivity, including altered auditory sensitivity, has recently been recognized as a core symptom of ASD under the new diagnostic criteria of DSM-5 [48]. In addition to evidence for auditory hypersensitivity revealed by clinical questionnaires [49, 50], recent behavioral experiments have revealed a more detailed picture of the altered auditory processing in ASD. Although individuals with ASD have been shown to be impaired in some auditory functions, such as auditory attention reorienting and the modulation of auditory perception [51], it is notable that they outperform TD subjects in a number of auditory functions, such as auditory stream segregation [52] and the perception of pitch [53] and time [54]. Abnormalities in sensations other than audition have been also reported in the previous literature [55, 56]. Although we acknowledge the need for further investigation to establish the functional significance of the volumetric enlargement of the right middle ventral sub-region, our observations raise the interesting possibility that this alteration may involve parts of the neural mechanisms underlying altered sensation in ASD.

### Limitations

We acknowledge a few limitations of the present study. Firstly, the present study restricted recruitment to adult males. This restriction was made because several studies have reported effects of age [57] and sex [58] on functional connectivity, at least for neurotypical populations. Given the possibility that age and sex may be critical factors for the etiological and phenotypic heterogeneity of ASD [59], we need to carry out further studies targeting specific populations excluded in the present study.

Secondly, we determined the optimal cluster number *k* by the combined use of VI and MI. However, the choice of the optimal *k* may be an inherent problem in any data-driven clustering analysis, given that the gold standard for the selection of this value has not yet been established. Indeed, previous studies have adopted a variety of methods for determining the optimal *k* value, such as using a combination of percent agreement and VI [19], VI only [36], validation indicator [17], and Dice’s coefficient between test and re-test data [33]. Among all of the possible measures, we selected MI and VI, because the combined use of the two measures allows for the estimation of the three critical features of clustering solutions: similarity, dissimilarity, and stability. Although we acknowledge some uncertainty in our analysis, we note that our method unequivocally determined the optimal *k* for each hemisphere and that the *k* = 8 was optimal even when the TD data was used (Additional file 4: Figure S4). These results indicate the validity and robustness of our method, at least for the current dataset.

Thirdly, in contrast to the task-related fMRI design, it may be argued that the resting-state FC study alone does not allow us to directly test hypotheses about the relationship between particular brain regions and their functions. To overcome this general limitation of the resting-state FC study, we have adopted the meta-analytical decoding method of Neurosynth, which uses meta-analysis of co-activation data in the fMRI literature and performs a reverse inference regarding the functions of the sub-region of interest from an FC pattern seeding that sub-region. Although this approach has been gaining credibility in recent fMRI studies [17, 60], we still acknowledge the need of the hypothesis-driven approaches, such as task-related fMRI studies, to establish the links between alterations in specific insular sub-regions and abnormal functions in the ASD brain. The present findings based on data-driven approaches are expected to provide specific hypotheses in designing such hypothesis-driven studies in the future.

Fourthly, only one control region was used to show that the functional alterations that we observed in the organization of the insula are not present in functionally intact brain regions in individuals with ASD. Although this remains a limitation of the study, at least in the intracalcarine cortex, we were able to show that the functional parcellation pattern in the ASD group was highly comparable to that of the TD group in visual inspection and in volumes of the corresponding sub-regions that were revealed using permutation test. Although more control regions may be needed, using the intracalcarine cortex as a control region has raised the specificity of our findings for the insula.

## Conclusions

To conclude, the present study uses data-driven clustering analysis of the multi-voxel resting state FC data to reveal altered functional parcellation in the insular cortex of the ASD brain. With the combined use of meta-analytic decoding analysis, we observed localized alterations in sub-regional organization in each side of the insula in ASD. In the left insula, two anterior sub-regions were merged into a single sub-region in which selective involvement in affective and emotional functions was absent. In the right insula, the middle ventral sub-region for auditory-related and sensory functions was significantly increased in volume. Such alterations in functional organization in the insular cortex may constitute neural abnormalities that underlie emotional/affective and sensory problems in ASD.

## Additional files

Additional file 1: Figure S1. The statistical test for between-group difference in the sub-regional volume using the permutation procedure. The histograms illustrate the null distribution of volume differences between groups (in voxels) induced by the permutation procedure in the left anterior dorsal (AD) sub-region (the anterior sector in ASD) (a) and in the right middle ventral (MV) sub-region (b). The red dashed vertical line indicates the observed volume differences between correctly labeled TD and ASD groups. The negative values indicate that the sub-region is smaller in TD brain compared to the corresponding sub-region in ASD. (PDF 650 kb)
Additional file 2: Figure S2. Determination of the optimal number of clusters based on VI and MI in intracalcarine cortex. The intracalcarine cortex was selected as a control region. The VI and MI values are shown for every clustering solution for *k* values ranging from 2 to 10. Arrows indicate either local minima of VI or local maxima of MI. Dashed lines denote the optimal number of solutions as determined using both VI and MI. The error bars denote standard errors of the mean for 100 repetitions of the split-half procedure (see the “Estimation of the optimal number of clusters” section). “n.s.” indicates no statistically significant difference between points. (PDF 334 kb)
Additional file 3: Figure S3. The patterns of functional parcellation in the intracalcarine cortex in TD and ASD. Figures show the parcellation patterns at the optimal cluster number (*k*) of 7. Each figure is presented in axial and magnified axial views. The color of each intracalcarine sub-region reflects the color of the corresponding sub-region in the TD and ASD groups. Note the highly comparable parcellation patterns between groups. (PDF 355 kb)
Additional file 4: Figure S4. VI and MI values for clustering solutions (*k* = 2 to 10) using only TD participants. The dashed line denotes the optimal number of solutions based on the same criteria as in Fig. 2. The error bars are standard errors of the mean. Each kind of arrows points to the optimal number of solutions based on “similarity” and “dissimilarity” criteria. “n.s.” indicates that the MI or VI values are not significantly different between the 2 bracketed points. (PDF 1125 kb)

## Abbreviations

AD: Anterior dorsal
ADOS: Autism Diagnostic Observation Schedule
AFNI: Analysis of Functional NeuroImages
AQ: Autism spectrum quotient
ASD: Autism spectrum disorder
AV: Anterior ventral
C: Center
DISCO: Diagnostic Interview for Social and Communicative Disorders
DSM: Diagnostic and Statistical Manual of Mental Disorders
DVARS: D referring temporal derivative and VARS referring the root mean squared
FC: Functional connectivity
FD: Framewise displacement
fMRI: Functional magnetic resonance imaging
GM: Gray matter
IQ: Intelligence quotient
JART: Japanese version of the National Adult Reading Test
MD: Middle dorsal
MI: Mutual information
MNI: Montreal Neurological Institute
MV: Middle ventral
PD: Posterior dorsal
PV: Posterior ventral
ROI: Region of interest
rs-fMRI: Resting-state functional magnetic resonance imaging
SD: Standard deviation
SPM: Statistical Parametric Mapping
TE: Echo time
TD: Typically developed
TR: Repetition time
VI: Variation of information
WAIS: Wechsler Adult Intelligence Scale
