# Supplementary figures and images for: Altered functional organization within the insular cortex in adult males with high-functioning autism spectrum disorder: evidence from connectivity-based parcellation
Source: Mol Autism. 2016 Oct 5;7:41. doi: 10.1186/s13229-016-0106-8 (PMC5052801; doi:10.1186/s13229-016-0106-8)

**a****AD (TD) vs Anterior section (ASD)**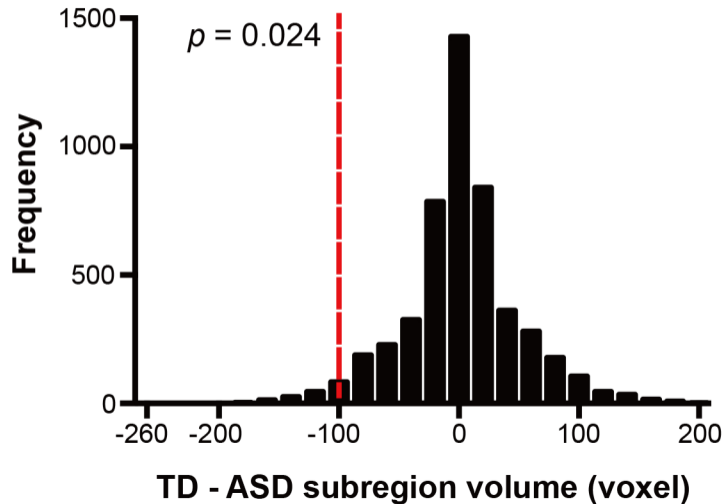**b****MV (TD) vs MV (ASD)**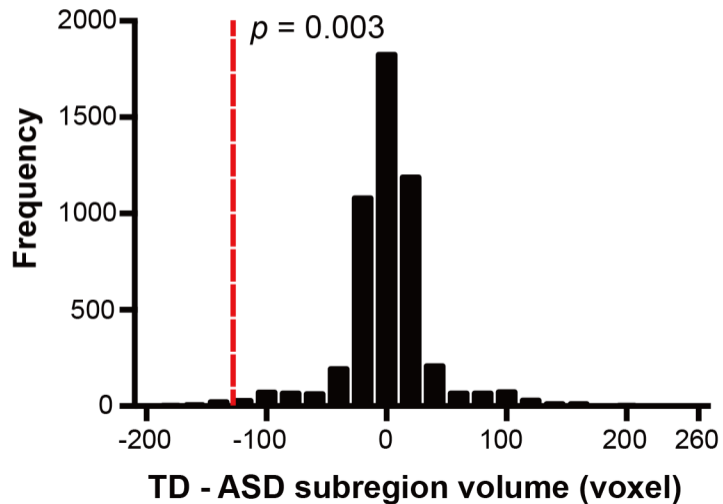

Supplement: Additional file 1: Figure S1. — The statistical test for between-group difference in the sub-regional volume using the permutation procedure. The histograms illustrate the null distribution of volume differences between groups (in voxels) induced by the permutation procedure in the left anterior dorsal (AD) sub-region (the anterior sector in ASD) (a) and in the right middle ventral (MV) sub-region (b). The red dashed vertical line indicates the observed volume differences between correctly labeled TD and ASD groups. The negative values indicate that the sub-region is smaller in TD brain compared to the corresponding sub-region in ASD. (PDF 650 kb) [file 13229_2016_106_MOESM1_ESM.pdf]

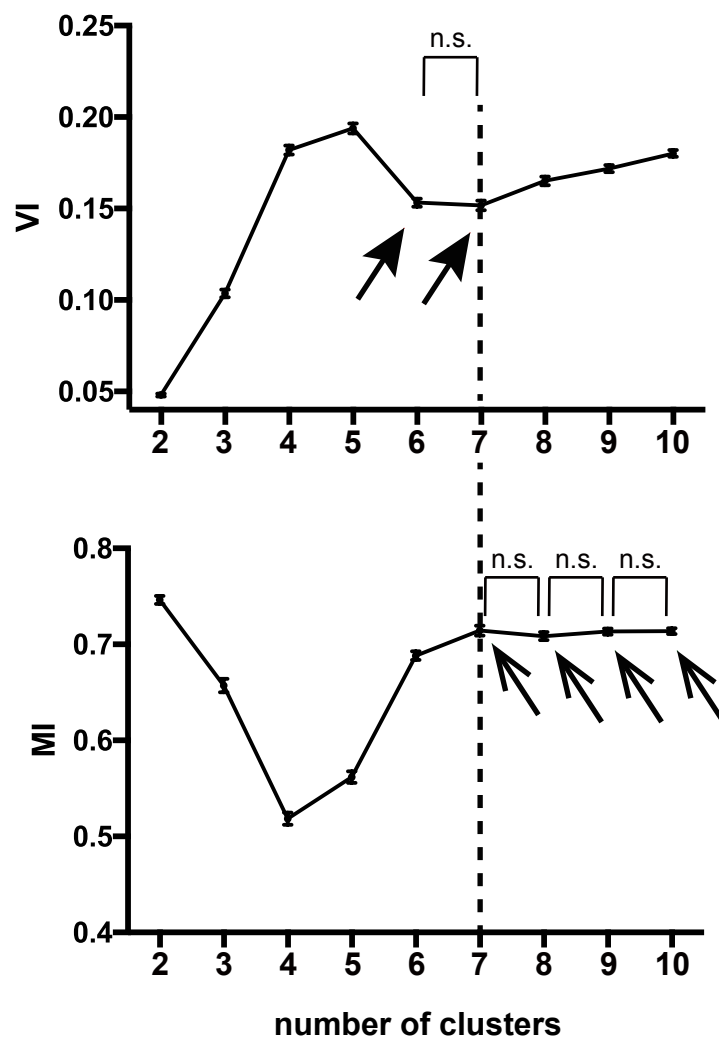

↗ Local minimum (VI)      ↖ Local maximum (MI)

Supplement: Additional file 2: Figure S2. — Determination of the optimal number of clusters based on VI and MI in intracalcarine cortex. The intracalcarine cortex was selected as a control region. The VI and MI values are shown for every clustering solution for k values ranging from 2 to 10. Arrows indicate either local minima of VI or local maxima of MI. Dashed lines denote the optimal number of solutions as determined using both VI and MI. The error bars denote standard errors of the mean for 100 repetitions of the split-half procedure (see the “Estimation of the optimal number of clusters” section). “n.s.” indicates no statistically significant difference between points. (PDF 334 kb) [file 13229_2016_106_MOESM2_ESM.pdf]

**a**

**TD**

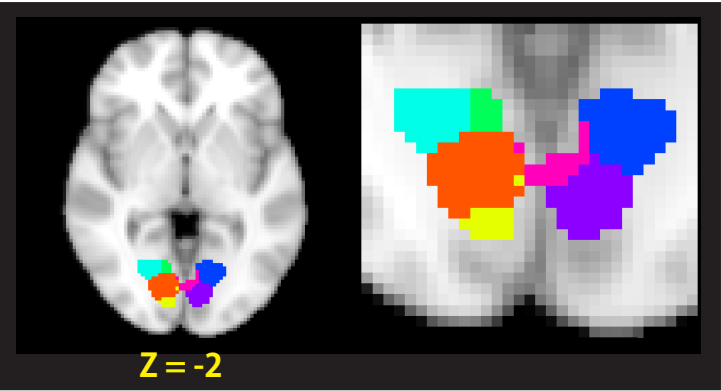

**b**

**ASD**

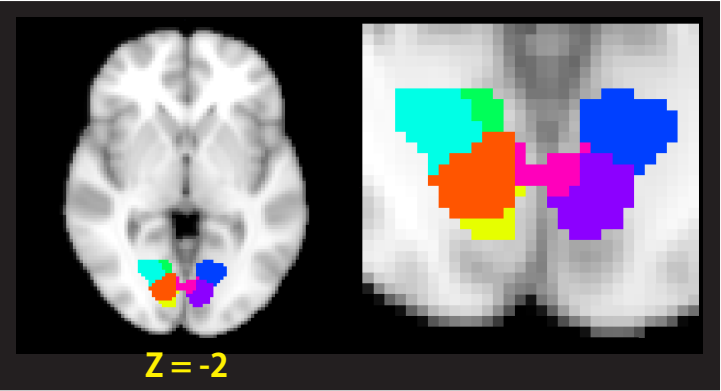

Supplement: Additional file 3: Figure S3. — The patterns of functional parcellation in the intracalcarine cortex in TD and ASD. Figures show the parcellation patterns at the optimal cluster number (k) of 7. Each figure is presented in axial and magnified axial views. The color of each intracalcarine sub-region reflects the color of the corresponding sub-region in the TD and ASD groups. Note the highly comparable parcellation patterns between groups. (PDF 355 kb) [file 13229_2016_106_MOESM3_ESM.pdf]

**a****Left insula**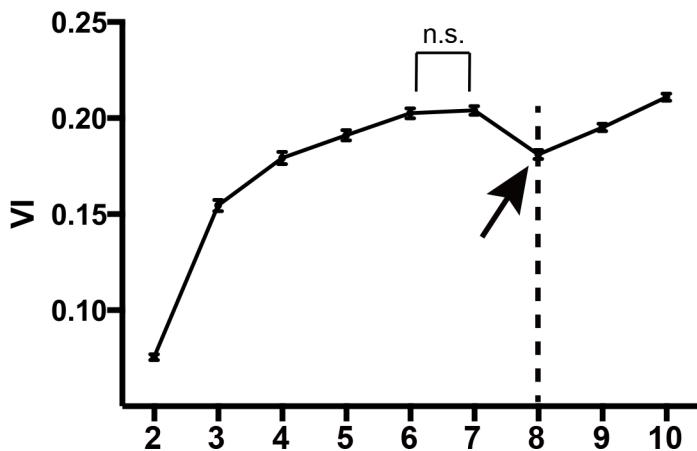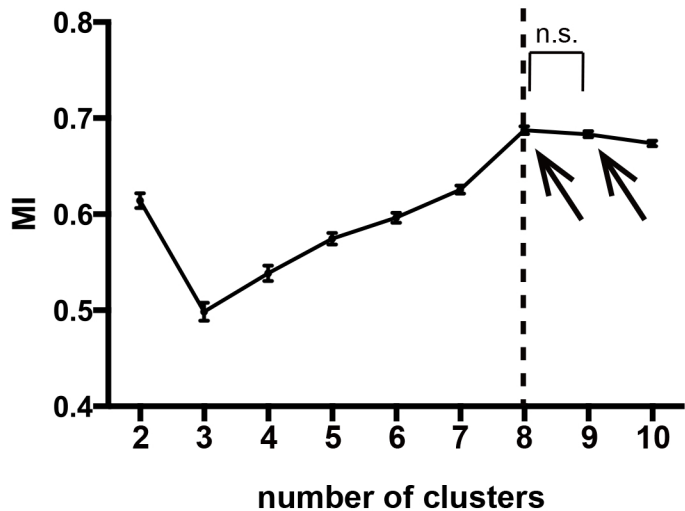**b****Right insula**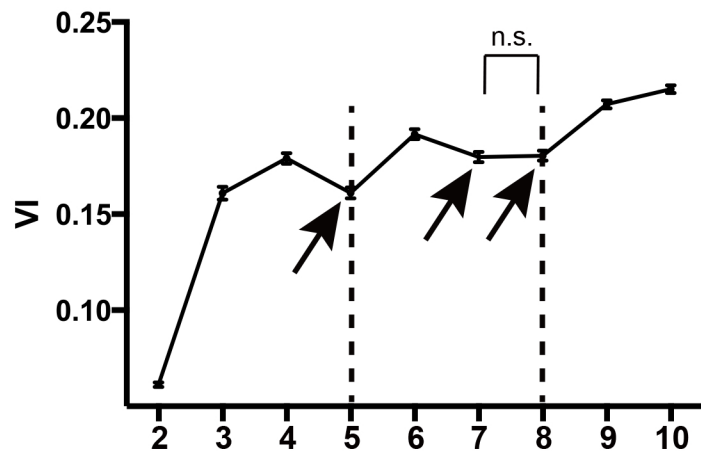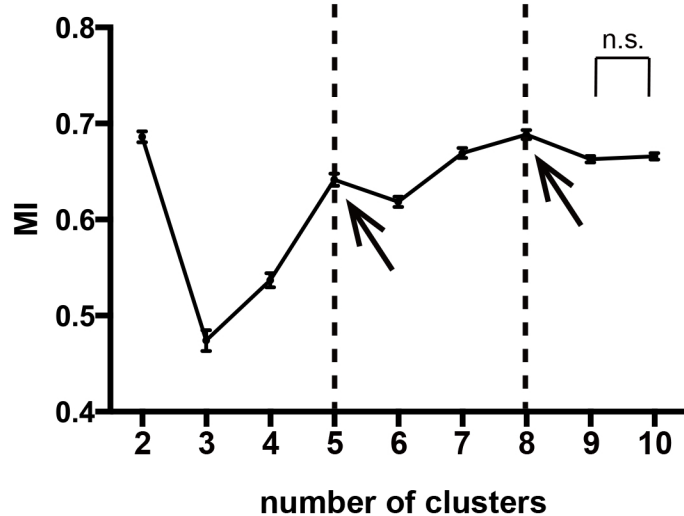

↗ Local minimum (VI)      ↖ Local maximum (MI)

Supplement: Additional file 4: Figure S4. — VI and MI values for clustering solutions (k = 2 to 10) using only TD participants. The dashed line denotes the optimal number of solutions based on the same criteria as in Fig. 2. The error bars are standard errors of the mean. Each kind of arrows points to the optimal number of solutions based on “similarity” and “dissimilarity” criteria. “n.s.” indicates that the MI or VI values are not significantly different between the 2 bracketed points. (PDF 1125 kb) [file 13229_2016_106_MOESM4_ESM.pdf]
